# Persistence and Culturability of *Escherichia coli* under Induced Toxin Expression

**DOI:** 10.3390/antibiotics13090863

**Published:** 2024-09-09

**Authors:** Yousr Dhaouadi, Mohamad Javad Hashemi, Dacheng Ren

**Affiliations:** 1 Department of Biomedical and Chemical Engineering, Syracuse University, Syracuse, NY 13244, USA; ydhaouad@syr.edu (Y.D.); mhashemi@syr.edu (M.J.H.); 2 BioInspired Institute, Syracuse University, Syracuse, NY 13244, USA; 3 Department of Civil and Environmental Engineering, Syracuse University, Syracuse, NY 13244, USA; 4 Department of Biology, Syracuse University, Syracuse, NY 13244, USA

**Keywords:** bacteria, persister, HipA, dormancy, VBNC, arabinose, ofloxacin

## Abstract

Background/Objectives: Bacteria are well known to enter dormancy under stress conditions. However, the mechanisms of different dormancy-related phenotypes are still under debate and many questions remain unanswered. This study aims to better understand the effects of toxin gene expression on the dormancy of *Escherichia coli*. Methods: The effects of toxin gene expression on growth, persistence, and culturability were characterized. Specifically, we detailed dose- and time-dependent dormancy of *E. coli* and its susceptibility to ofloxacin via arabinose-induced *hipA* toxin gene expression under the *P_BAD_* promoter. A new plot was developed to better describe the dynamic changes in culturability and persistence. The expression level of *hipA* was determined using qPCR and cellular activities were monitored using fluorescence imaging and flow cytometry. Results: High-level persister formation and strong tolerance to ofloxacin were observed after high-level *hipA* induction. The new plot reveals more information than the changes in persistence alone, e.g., reduced culturability of *E. coli* and thus deeper dormancy under high-level *hipA* induction. Consistently, controlled *hipA* induction led to decreased cellular activities at promoter *P_rrnBP1_* and an increase in the non-culturable subpopulation. Conclusions: Overall, this study provides new insights into dormancy induced by toxin gene expression and a more comprehensive view of persistence and culturability. The findings may help develop better control agents against dormant bacterial cells.

## 1. Introduction

Bacteria are well known to survive the attack of antibiotics by entering dormant states [1]. Conventional antibiotics were developed based on growth inhibitory activities; thus, most agents are only effective against metabolically active cells [2]. Understanding the intricate mechanisms governing bacterial antibiotic tolerance is of paramount importance for research on chronic infections and the development of more effective treatment strategies [3]. Antibiotic tolerance is based on phenotypic changes in bacterial cells, which is different than resistance that evolves through genetic mutations or the acquisition of resistance genes [4]. However, research has shown that persistent bacteria may be more prone to developing antibiotic resistance over time than susceptible counterparts [3,5], creating further challenges to treating infections. Thus, new knowledge and control strategies are needed to address the challenges of antibiotic tolerance.

Two key dormant states that have garnered increasing attention are the formation of persister cells and viable but non-culturable cells (VBNCs). Both persister cells and VBNCs exist in quiescent states and are highly tolerant to conventional antibiotics. However, there are also major differences between them. Persister cells can regrow after the stressor is removed [6,7]. In contrast, VBNCs have lost culturability in the original medium that they are formed in but can be resuscitated in a different medium, and some resuscitation factors may be needed [8,9]. There have been significant debates and controversies about the nature of these two phenotypes and the mechanisms of their formation [10,11,12]. It has been proposed that there is a continuum between active cells and cell death, with VBNCs being at a deeper state of dormancy than persister cells [12]. There are also studies showing that VBNCs are actually dead cells [10] or possess low metabolic activities [13,14], as well as proposals of different terms for these subpopulations [15]. Clearly, more studies are needed to fully understand these important states of bacterial response to environmental stresses.

Persister cells are of particular importance because surviving persisters can reestablish the population after the stress is withdrawn [16,17]. Environmental factors play a significant role in bacterial persistence, shaping their ability to survive and adapt [18]. Bacteria exhibit varying degrees of persistence in response to factors such as temperature, pH, nutrient availability, and oxygen levels [19]. In host environments, the interplay with the immune system and biofilm formation can contribute to chronic infections [20,21]; and both fungal and bacterial cells have been shown to persist inside macrophages [22,23]. Understanding how cells first become tolerant to harsh stressors is crucial for developing strategies to mitigate antibiotic tolerance and control chronic infections.

The study of persistence involves investigating mechanisms that allow bacteria to enter and exit the dormant state of reduced metabolic activity. The formation of persisters typically involves stressors such as low nutrient levels, high osmolarity, pretreatment with protein synthesis inhibitors or overexpression of a toxin gene [6,24,25]. In this study, we focus on persister formation through overexpression of the toxin gene *hipA*, which drastically increases persister fractions in *E. coli* [26]. It is part of the *hipBA* Type II toxin antitoxin (TA) module [27], and ectopic expression of *hipA* in *E. coli* leads to significant growth inhibition due to glutamate starvation and stalled ribosomes [28]. Intracellularly, HipA is bound to the antitoxin HipB and only found in the free form after protease degradation of HipB to free HipA [27,29]. In this study, we quantitatively followed *E. coli* persister formation and susceptibility to ofloxacin challenge through arabinose-induced *hipA* expression from the *P_BAD_* promoter. Previous studies on *hipA* and persistence are largely based on optical density measurements and short-term induction of planktonic cells [30,31,32,33,34,35]. Single-cell studies have also been conducted with short-term *hipA* induction and showed that *hipA* reduces growth at thresholds of expression to yield a mixed population of dormant and normal cells, emphasizing that the HipBA module is a bet-hedging strategy of adaptive evolution [36].

More research is needed to elucidate the dynamic change of culturability with long-term *hipA* induction, as persistence but not VBNC following toxin gene overexpression has been the focus of previous studies. Focusing on the changes in persistence and culturability with prolonged time and dose-dependent arabinose induction, this study aims to unravel the dynamics of persister formation and changes in culturability under ectopic expression of the toxin gene *hipA*. Using a new plot of persistence vs. culturability, more insights into the dynamics of dormancy were obtained by varying the level of *hipA* induction. Better understanding of the dynamics of persistence carries significant implications for therapeutic strategies and experimental designs, which could guide drug development to effectively target dormant cells.

## 2. Results

### 2.1. Dynamic Changes in Persistence and Culturability under hipA Expression

**Figure 1:**
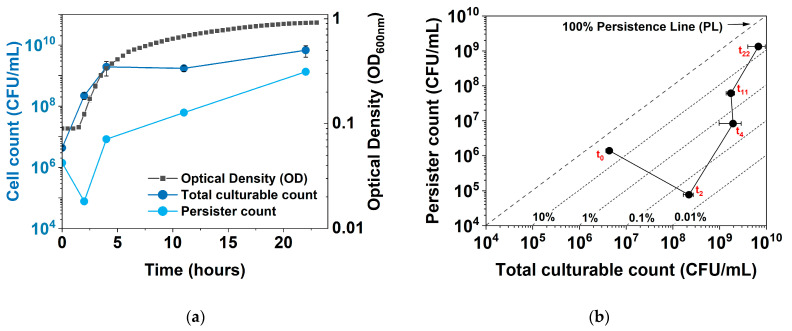
Growth, persistence, and culturability of *E. coli* BW25113/pRJW1. (**a**) Conventional plot showing OD_600_, viable cell count (total CFUs), and persister count during a 24-h time period. Means ± SE are shown (n = 3). (**b**) New plot showing the changes in culturable cell count and persister cell number during the same culturing period, with persistence lines (PL) of survival after ofloxacin challenge. Means ± SE are shown (n = 3).

To obtain more insights into the dynamic shift between culturability, growth, persistence, and dormancy, we developed a new plot to follow persistence vs. culturability of *E. coli* during normal growth and under *hipA* induction. Figure 1 shows the comparison of a conventional plot of persistence vs. time and the new plot of persister count vs. culturability (total CFUs). Unlike the conventional plot, which shows an increase in persistence with a plateau in stationary phase, the new plot shows a “V” shape, with an initial increase in total CFUs (cell growth) and a decrease in persister cell count, followed by a sharp increase in persistence. In addition, diagonal persistence lines (PLs) of a specific persistence level, e.g., 1%, 10%, or 100%, can be added to allow direct comparison with the conventional plot. We name this new plot the Persistence–Culturability plot (PC plot).

Moreover, the new PC plot was found to be particularly helpful when there is a significant subpopulation of nonculturable cells (VBNCs). In principle, persister percentage is calculated per Equation (1):

(1) $$\mathrm{Persister}\;\mathrm{percentage}\;(\%)=\frac{\mathrm{Number}\;\mathrm{of}\;\mathrm{persister}\;\mathrm{cells}}{\mathrm{Total}\;\mathrm{CFU}+\mathrm{Number}\;\mathrm{of}\;\mathrm{VBNCs}}\times 100\%$$

Because the number of VBNCs is usually unknown, the persister percentage based on CFUs can be incorrect and misleading. For example, if there is a shift towards deeper dormancy or cell death, the number of persister cells will decrease. But if it decreases more than the total CFUs, the persister percentage will decrease. This will be misleading, as the population is entering a deeper dormancy state (with reduced culturability), but the persistence is seemingly decreasing.

Such changes can be better captured in the new PC plot by comparing the direction that the data points are shifting to. Specifically, the move towards the lower right corner indicates growth and reduced dormancy. Moving towards the upper right indicates growth and associated persister formation, e.g., in the stationary phase of culturing or under certain stress. There are also moves to the left that are not reflected in conventional plots. For example, moving to the upper left indicates a decrease in total culturable counts and increases in both persister formation and possibly VBNCs. The move towards the lower left, on the other hand, indicates a decrease in both total culturable counts and persister cells. This suggests that the population is entering a deeper dormancy, with significant VBNC formation and even cell death.

**Figure 2:**
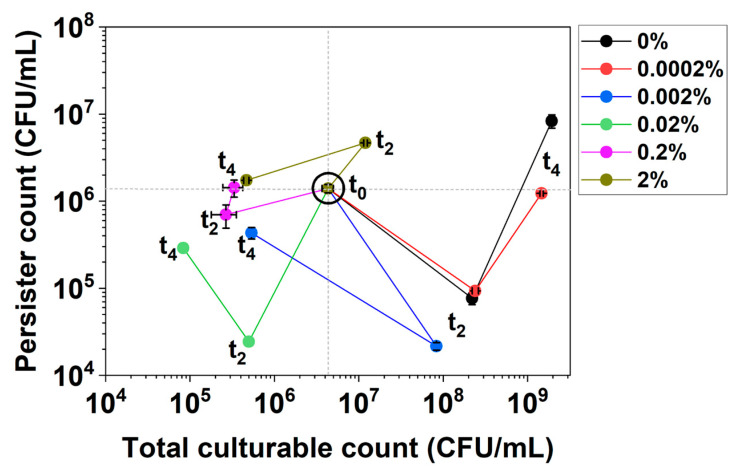
Persistence–Culturability (PC) plot of *E. coli* BW25113/pRJW1 during early culturing under *hipA* induction. Different moving directions of the plot indicate complex dynamics of growth and dormancy under different levels of induced toxin production by arabinose. Means ± SE are shown (n = 3).

These changes can be illustrated by the first 4 h of culturing of *E. coli* BW25113/pRJW1 under *hipA* expression (Figure 2). The experiment was carried out in the same manner as the control in Figure 1b, but the cells were induced with different concentrations of arabinose. For low-level induction with 0.0002% arabinose and uninduced control, the culture showed significant growth, with persistence decreasing first and then increasing during growth (V-shape curve). With higher concentrations of the inducer arabinose, however, the cultures exhibited decreased culturability and changes in persistence that moved the curves to the lower or upper left quadrants (e.g., under 0.02% arabinose induction).

**Figure 3:**
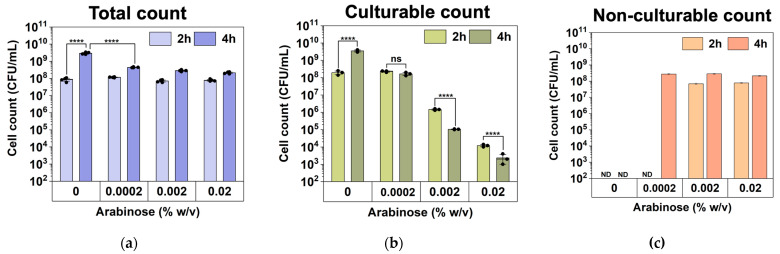
Culturability during the early stage of culturing under *hipA* induction (2 and 4 h after inoculation). (**a**) Total cell numbers based on counting chamber results. (**b**) Culturable counts based on CFU assay. (**c**) Total number of non-culturable cells. Means ± SE are shown (n = 3). Stars indicate the *p* values, **** *p* < 0.0001. “ns” indicates no significance. “ND” means not detected.

**Figure 4:**
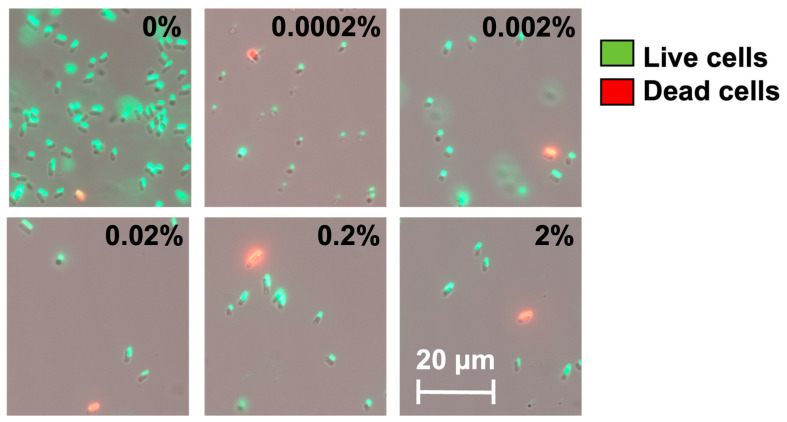
Live/Dead staining of cells after 5 h incubation with or without arabinose induction. The percentage marked in each image indicates the amount of arabinose. Representative images from 4 biological repeats are shown.

We speculate that these high-level induction conditions may lead to VBNC formation. To understand this, we used a counting chamber to quantify the total number of cells after *hipA* induction and compared that with CFU counts to determine the number of non-culturable cells (difference between the two numbers), as described previously [37]. The experimental conditions were the same as those in Figure 2 except that the cultures were grown in culture tubes with a larger volume (2 mL). As shown in Figure 3, the total number of cells did not change significantly after 2 h of incubation among the induced conditions. However, the number of culturable cells decreased significantly with *hipA* expression, and the decrease was positively correlated with the concentration of the inducer arabinose. Consistently, large subpopulations of non-culturable cells (difference between total cell count and culturable count) were observed within 2 h with 0.002% or 0.02% arabinose induction, which required an additional 4 h with a weaker 0.0002% induction. This is consistent with the threshold-based persistence induced by *hipA* expression [36]. Cell death was negligible based on Live/Dead staining after 5 h of culturing (Figure 4; despite a minor shift in cell position in the overlay when changing between fluorescent and brightfield channels during imaging). This indicates significant VBNC formation under these stress conditions.

### 2.2. Dynamic Changes in Culturability and Dormancy under hipA Induction

**Figure 5:**
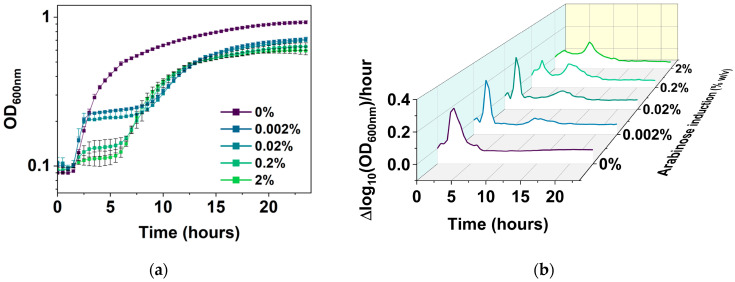
Growth of *E. coli* BW25113/pRJW1 under different levels of *hipA* induction. (**a**) Growth curves based on OD_600_. Means ± SE are shown (n = 4). (**b**) Growth rates (means of 4 biological repeats).

The new PC plot was then used to follow *E. coli* BW25113/pRJW1 cultures over a 24 h period without or with 0.0002%, 0.002%, 0.02%, 0.2%, or 2% arabinose induction, and compared with conventional plots of cell growth based on OD_600_ and CFU. Unlike the uninduced control, which had typical growth with lag, exponential, and stationary phases (Figure 5a), the induced cultures exhibited a biphasic growth pattern with a second exponential phase after the first stationary phase. Induction of *hipA* showed stronger effects on the growth rate in the second growth phase than the first phase (Figure 5b). The behavior of regrowth despite active toxin induction has been noted by others in the field [34] and is likely due to the autophosphorylation of HipA that inhibits its kinase activity, and thus reduces the copy number of active HipA [38]. The present study provides more details and a comprehensive visualization of the kinetics under different levels of *hipA* induction.

It is notable that the time taken to reach a second exponential phase is inversely correlated with the arabinose concentration. This is consistent with the possible effects of HipA autophosphorylation as the abundance of free HipA changes [38]. With 0.002% arabinose induction, cells exhibited an initial jump in growth around 2 h of subculture before a stationary phase from 3 h to 7 h after inoculation. Cells resumed a slower growth at the 10 h mark at lower induction levels of 0.002% and 0.02%. Stronger inductions resumed growth earlier as the strength of the *hipA* induction increased, e.g., between 5 and 7 h for 0.2 and 2% arabinose induction, and exhibited a much shorter initial exponential phase.

**Figure 6:**
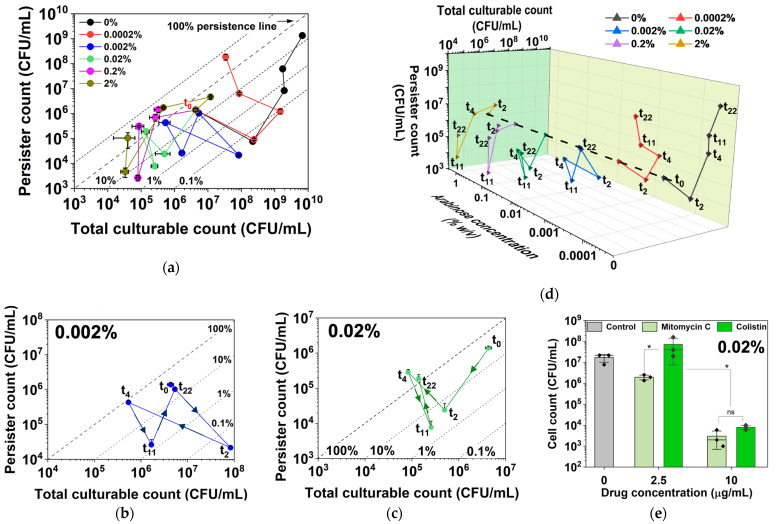
Persistence and culturability under different levels of *hipA* induction. (**a**) Number of persister cells and total CFU with and without *hipA* induction. (**b**,**c**) Zoomed-in plots for 0.002% (**b**) and 0.02% (**c**) induction are included to illustrate various movements across the quadrants under these more dynamic conditions. (**d**) A 3D plot of the same data to better reveal the dynamic changes in culturability and persister cell count. The expression of *hipA* was induced by 0.0002%, 0.002%, 0.02%, 0.2%, or 2% arabinose. Persister cells were isolated by treating with 10 µg/mL ofloxacin for 5 h in PBS. Means ± SE are shown (n = 3). (**e**) Persister killing assay conducted with mitomycin C and colistin treatments for 1 h in PBS. Persisters were formed with 0.02% arabinose induction for 4 h. Means ± SE of culturable cell counts are shown (n = 3). Stars indicate *p* values, * *p* < 0.05, “ns” indicates no significance.

The plot of persister cell count vs. total culturable count revealed more information about cell growth and dormancy under these conditions. The percentage of persister cells out of the total culturable cells can be estimated based on the location of the data point relative to the persistence lines of 100%, 10%, 1%, and so on (Figure 6a). As seen in Figure 2, the uninduced control and 0.0002% arabinose conditions experienced an increase in the culturable cell count and a decrease in the persister count over the first 2 h, causing the curve to move towards the lower right. When the uninduced culture reached the stationary phase, the increase in total number of culturable cells slowed down, while the persister number increased sharply. The transition to the upper right quadrant of the curve indicates growth-related persister formation. For cultures induced with 0.0002% arabinose, the curve bent to the upper left direction by 11 h, indicating VBNC formation along with an increase in persistence (Figure 6a,d).

Interestingly, the curves in Figure 6a were found to move to the left earlier on for induction with 0.002% or higher concentrations of arabinose, which indicates decreases in culturability and the formation of VBNCs and potential cell death. The trends under 0.002% and 0.02% in Figure 6b and Figure 6c, respectively, are zoomed-in views to illustrate the various quadrant movements under these dynamic conditions. At 0.002%, cells showed an increase in culturability only within the first 2 h before increases in persistence and possibly VBNC occurred by 4 h. The cells eventually overcame the active induction and resumed culturability along with increasing persistence by 22 h. In contrast, 0.02% induction exposed the cells to a higher level of toxin, and cells exhibited rapid decreases in both culturability and persistence within 2 h, indicating possible VBNC formation. To validate the arabinose-induced *hipA* persister cells, a condition that yielded ~100% persistence (4 h of culturing with 0.02% arabinose) was used to harvest persister cells for treatment with mitomycin C and colistin, both of which are known to kill antibiotic-isolated persisters [39]. The results showed dose-dependent killing of arabinose-induced *hipA* persisters by both agents (Figure 6e).

For 0.2% and 2% inductions, the total culturable count decreased by much more than the persister count over the first 4 h before the cells eventually settled at 100% persistence by 22 h, with the lowest culturable count observed in this assay. This graphing method allows for better visualization of the data by showing both absolute numbers and relative percentages, providing a comprehensive view of dormancy trends and enabling comparative analysis across the different induction strengths.

### 2.3. Dynamic Changes in Cell Counts Revealed More Information about Dormancy

The comprehensive PC plot of persister count, total culturable count, and persister percentage was used to further evaluate the dormancy of *E. coli* BW25113/pRJW1 under *hipA* induction by different concentrations of arabinose. As expected, induced *hipA* expression slowed down the growth and caused significant persister formation. Induction with 0.0002% arabinose reduced viable counts during the early stage of culturing, but cells could still reach close to stationary-phase CFU counts as the uninduced control before the curves bent to the left quadrant (Figure 6a). At 0.002% induction and above, the typical V shape of the PC curve was no longer observed. This indicates the dynamic changes in the HipA pool and the shift between active cells, persisters, and VBNCs best visualized in the 3D plot (Figure 6d). Overall, induction of *hipA* moved curves to the left in a dose-dependent manner. Importantly, ~100% persistence to ofloxacin (persister count vs. total CFUs) was reached under specific conditions: 0.0002% for 22 h; 0.002% for 4 h; 0.02% induction for 4 and 22 h; 0.2% induction for 2, 4 and 22 h; and 2% induction for 4 and 22 h.

It is interesting to note that with a 0.02% arabinose induction, cells reached the highest level of dormancy within 4 h of induction and then fell to 3% by 11 h before the persister cell count began to increase again. Complex changes were also observed for some other conditions, e.g., increases in persister count with stable culturable count occurred with 0.2% and 2% arabinose induction. Because 100 µg/mL ampicillin was present in the culture medium, the overcoming of dormancy was not due to loss of the plasmid carrying *hipA*, but likely because of HipA autophosphorylation [38], as has been reported to occur with HipA overexpression [34].

### 2.4. Arabinose-Induced Expression of hipA

**Figure 7:**
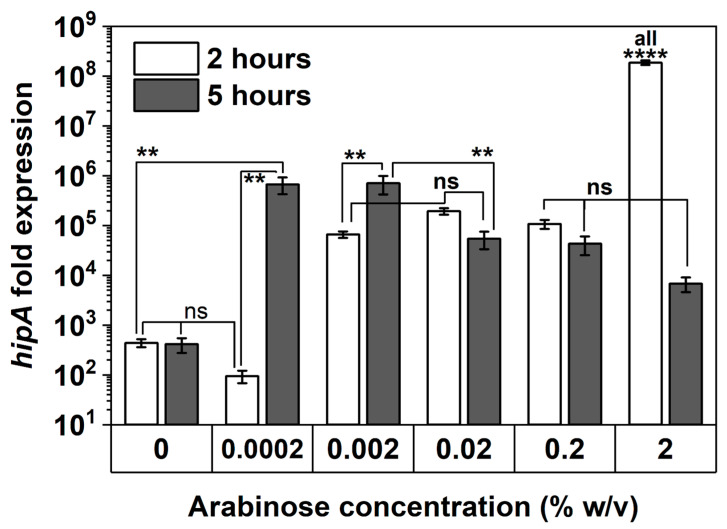
*hipA* expression with and without arabinose induction. The mRNA level of *hipA* relative to the empty vector control was quantified by qPCR after 2 and 5 h of culturing time. Housekeeping gene *rrsA* was used as a control for baseline expression. Means ± SE are shown (n = 3). Stars indicate the *p* values, **** *p* < 0.0001, ** *p* < 0.01, “ns” indicates no significance.

To corroborate persister assay results, the transcriptional levels of *hipA* in *E. coli* BW25113/pRJW1 after 2 and 5 h of culturing were evaluated using qPCR (Figure 7). With 2 h of induction, *hipA* expression was found to be positively correlated with the concentration of arabinose, particularly at the highest 2% induction within 2 h of culturing (*p* < 0.0001). The level of *hipA* remained unchanged from 2 to 5 h for uninduced control and increased significantly for 0.0002% and 0.002% inductions during the same period of time (*p* < 0.01). In comparison, the level of transcription did not increase further for 0.02% or 0.2% arabinose induction, indicating that a threshold was reached. With 2% induction, the level of *hipA* was found to be lower at 5 h than at 2 h. This decrease in mRNA level of *hipA* indicates that long-term high-level induction may reduce the pool of active HipA and therefore allows the cells to enter the second stage of dormancy, which occurred as early as the 5 h mark, per Figure 5a. This is consistent with previous reports of repeated cycling of HipA [34], which can be caused by autophosphorylation of HipA that is inactivated when induced at high levels [38]. The absence of an increase in *hipA* mRNA level over time with 0.02, 0.2, and 2% arabinose induction is consistent with the overcoming of dormancy at these thresholds, per Section 2.3.

### 2.5. Corroborating the Results Using a Growth Reporter Strain

**Figure 8:**
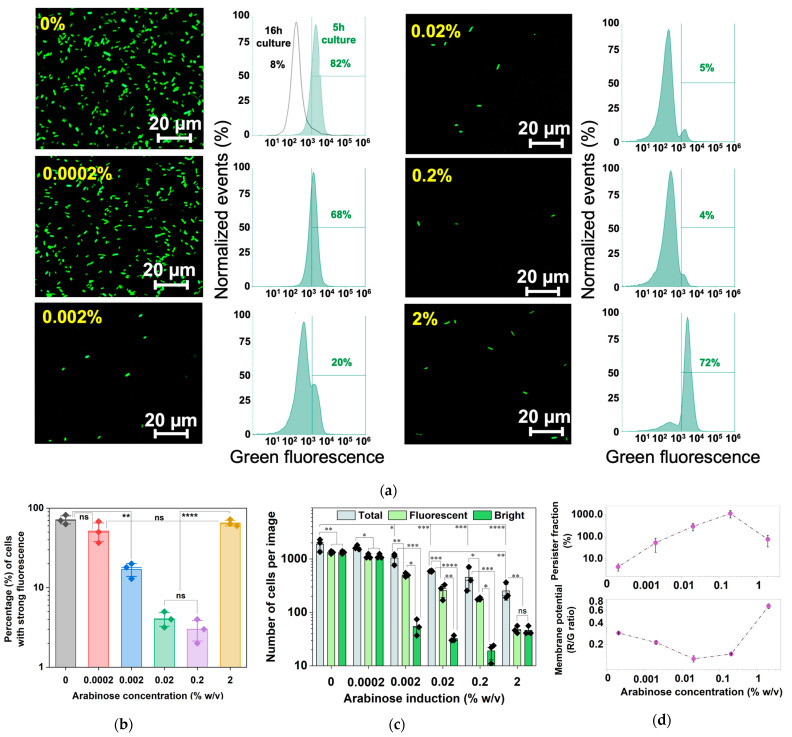
Persister formation, growth rate, and cell count of arabinose-induced *E. coli* BW25113 *P_rrnB_*_P1_-GFP_AGA_/pRJW1. (**a**) Fluorescence images of dose-dependent 5 h arabinose induction along with fluorescence quantified with flow cytometry. (**b**) A plot of bright cell quantification from flow cytometry of *E. coli* BW25113 *P_rrnB_*_P1_-GFP_AGA_/pRJW1 fluorescence after 5 h of arabinose induction. Means ± SE are shown (n = 3). (**c**) The total, bright, and fluorescent (bright and dim) cell counts were determined based on the images in (**a**) using ImageJ version 1.53k. Means ± SE are shown (n = 3). (**d**) Membrane potential was determined after 5 h dose-dependent arabinose induction along with persister levels after ofloxacin challenge. Means ± SE are shown (n = 3). Stars indicate the *p* values, * *p* < 0.05, ** *p* < 0.01, *** *p* < 0.001, **** *p* < 0.0001, “ns” indicates no significance.

To further corroborate the results, a reporter with unstable GFP_AGA_ [40] under the promoter *P_rrnB_*_P1_ was constructed and tested. After 5 h of induction at 37 °C, a decrease in fluorescent cells was observed as the arabinose concentration increased, consistent with increasing dormancy and presumably less production of ribosomal subunits for growth activities at *P_rrnB_*_P1_. Consistently, the results of flow cytometry indicate that 0.2% arabinose induction yielded the lowest fraction (4% ± 2%) of fluorescent cells and thus high-level dormancy. Interestingly, we found an increase in the fluorescent signal at 2% arabinose induction compared to 0.2%. In general, increasing the induction strength of *hipA* yielded a significant reduction in both fluorescence and total cell counts. At 2%, however, despite the total cell count being at the lowest relative to weaker inductions, almost all cells had strong fluorescence, indicating that dormancy was not the dominating phenotype under this condition (Figure 8). This is consistent with cell counts and the mRNA results, and the possible effects of autophosphorylation of HipA rescuing the cell from a latent state.

The results from 5 h cultures showed that as the percentage of survival to ofloxacin increased, the membrane potential decreased, reaching a minimum value with 0.2% arabinose induction, consistent with the dormancy phenotype for antibiotic tolerance (Figure 8d). For induction with 2% arabinose, the red/green signal ratio increased rapidly, implying that cells in this state were hyperpolarized. This active signal followed the same trend as the increased reporter strain signal shown in Figure 8b. These findings indicate that cellular activities decreased as arabinose concentration increased, up until a threshold of between 0.2% and 2%. At 2%, the signal increased instead.

To understand the higher fluorescence under 2% arabinose induction, we quantified the brightfield and fluorescence images (n = 3) using ImageJ for each concentration of arabinose induction (Figure 8c). The results show that increasing induction of *hipA* yielded a significant reduction in total fluorescence, and the total cell count in brightfield images also decreased significantly. There was also a significant population of dim cells among inductions with 0.002%, 0.02% and 0.2% arabinose. At 2%, however, despite the total cell count being at the lowest relative to weaker inductions (*p* < 0.0001), the bright cells equated the total fluorescent cell count, indicating that there were negligible dim cells. This is consistent with the results in Figure 5b, which show a shorter time before entering the second growth phase under 2% arabinose induction compared to other conditions.

## 3. Discussion

Previous studies commonly report the level of persistence as the percentage of persister cells vs. the total culturable cell count, e.g., the number of persisters isolated with a specific stressor (e.g., an antibiotic) divided by the total CFUs of the sample at selected timepoints of treatment. The CFU number is determined after plating cells on an agar plate, usually with the same culture medium, and is commonly viewed as a valid representation of viability. However, the CFU assay requires cell growth. Thus, it only represents culturability, not viability [41]. It has been reported that bacteria may remain viable but lose culturability under stress, e.g., entering the viable but non-culturable state known as VBNC [42]. These cells are still viable but would not be included in the total CFUs if the right condition for resuscitation is not identified or used in the CFU assay. Thus, using total CFUs may not give an accurate estimate of persistence level if there is significant VBNC formation, especially if other factors can change cell viability during persister isolation (e.g., long or repeated incubation with antibiotics). There are published protocols for quantification of VBNCs through culturing and staining procedures [37], using PCR to differentiate dead cells [43], and studying VBNCs with TEM imaging [44]. However, these methods are not high throughput and there are debates about the effectiveness/accuracy of current methods [10,15,44]. The dynamic changes in the populations of VBNCs, persister cells, and dead cells under stresses are not fully understood. Thus, it is important to present results with the full information of persistence and culturability when there is not enough information to determine viability.

This study highlights the heterogeneity in bacterial populations under stress conditions. The results and new PC plot reveal a distinct pattern of persistence at specific induction levels. In planktonic cultures of *E. coli* BW25113/pRJW1, cells at inoculation (t_0_) were tolerant to ofloxacin—akin dilution from the stationary phase. By plotting the data as persister count vs. total culturable count, the plot revealed dynamic changes in culturability and persistence during culturing and the effects of induced toxin HipA expression. The move in specific directions and quadrants reveals more information about dormancy, e.g., moving to the lower left quadrant indicates possible VBNC formation and/or cell death. Under high-level inductions with 0.02%, 0.2%, and 2% arabinose, the culturability declined after inoculation. Because no major cell death was observed based on PI staining (Figure 4), the decrease in culturability may be attributed to deeper dormancy and the formation of VBNCs. For example, with 0.2% arabinose induction, the total culturable cell count decreased by 1.1 logs in 4 h, with no decrease in persister count. This indicates that a significant fraction of cells lost culturability to enter the VBNC stage. This continued for 11 h after inoculation, with another 0.5 log decrease in culturable count, along with a 2.8 log decrease in persister count, before an increase in persistence occurred, moving the curve straight up (Figure 6).

It is interesting that under certain stress conditions, e.g., with high-level *hipA* induction by 2% arabinose, cells resumed growth after a period of dormancy. This led to a population of growing cells with higher membrane potential signals (Figure 8d) and a fraction of fluorescent cells seen at GFP tagged as *P_rrnB_*_P1_-GFP_AGA_ (Figure 8b,c). These results provide new information about bacterial culturability and dormancy under long-term induction of toxin gene expression. In Figure 5a, cells under 0.2% and 2% arabinose induction began entering the second exponential phase at the 5 h mark, while other conditions were in the lag between exponential phases 1 and 2, of which phase 1 dominated the overall growth rate in Figure 5b. The highest survival to ofloxacin was also observed when the *P_rrnB_*_P1_ signal was at its lowest (Figure 8d). Therefore, it is important to consider the strength and duration of stress exposure for its effect on the culturability of surviving viable persisters.

An explanation for the re-emergence of cell growth under high-level *hipA* induction is the autophosphorylative activity of HipA, a serine–threonine kinase that primarily phosphorylates glutamyl-tRNA synthetase GltX and causes persister formation [30,32]. Recent studies showed that HipA also phosphorylates other proteins, including itself [30,38]. The autophosphorylation leads to inactivation of kinase activity and can reduce the pool of functional HipA and eventually take the cells out of dormancy [38]. It will be interesting to quantify whether there is a threshold level of HipA dormancy that allows for such a reversion. Finer levels of controlled induction and comparison with toxins that do not have such auto-phosphorylation would also help in understanding the underlying mechanism. This is part of our future work.

It is worth noting that some *E. coli* clinical isolates from patients with chronic urinary tract infections carry the *hipA7* mutation [26]. HipA7 differs from HipA in substitutions G22S and D291A, which lead to a weaker binding with the antitoxin HipB, thereby increasing the concentration of free HipA7 relative to wildtype HipA [31]. Although HipA7 was found to increase persister fractions by 10–100 times more than the wildtype HipA, it was found to be nontoxic to the cells [45], implying that the ability to increase persister fractions could be distinct from the ability to induce dormancy [30,31]. Additionally, dormancy studied as a result of *hipA7* exposure may not reflect the mechanisms of active toxins of TA systems [46]. Semanjski et al. [30] performed phospho-proteomic studies after induction of *hipA* and *hipA7* from the arabinose-inducible promoter *P_BAD_* with 0.2% arabinose. The authors found that both *hipA* and *hipA7* targeted GltX as their main substrate for phosphorylation, in line with current mechanistic understanding of the protein, but HipA7 is a weaker kinase and does not phosphorylate as many targets as HipA. HipA exhibited strong kinase activity across a diverse pool of substrates controlling replication, transcription, and translation, as well as membrane transport proteins OmpA and OmpC [30]. Outer membrane proteins are commonly found to play a role in persistence, with OmpF identified as vital for post-antibiotic recovery in the resuscitation of depolarized persisters [47]. It will be interesting to test the *hipA7* induction and compare with the results in this study.

Bacteria are well known for their capability to enter dormancy and survive harsh conditions [48]. Both persister cells and VBNCs can tolerate high concentrations of antibiotics, and both have significance in infectious disease. However, the relationship between these two phenotypes is still being debated [49]. Ayrapetyan et al. [12] described that there is a continuum between live cells to death of bacteria with VBNCs as a deeper stage of dormancy than persister cells. In contrast, there have also been reports that VBNCs are actually dead cells with empty shells [10,50], and some argue that VBNCs are live cells with active yet low metabolic activities [11,51,52]. Some of these discrepancies may be due to the lack of effective methods to isolate these cells and maintain them in their physiological stage. It is generally accepted that VBNCs have lost culturability in the medium of the original culture and that some require specific factors to resuscitate [8,9]. Thus, there is a challenge in differentiating viable cells, persister cells, VBNCs, and dead cells, especially the dynamic changes during culturing. We believe the new PC plot described in this study can provide helpful insights into the dynamic changes between these physiological stages of bacterial cells. Future studies of different bacteria and stressors will help further develop such analysis to decipher the bacterial dormancy spectrum and develop more effective control methods.

## 4. Materials and Methods

Bacterial strains and culturing conditions. The plasmid pRJW1 was cloned by insertion of wildtype *hipA* sequence into the *P_BAD_*/*hisD* vector (ThermoFisher, Waltham, MA, USA) using multiple cloning sites HindIII and NdeI via traditional restriction–ligation cloning and heat-shock transformation. Ampicillin at 100 μg/mL was used to select the correct colonies. The host strain used in this study was *Escherichia coli* BW25113 (CGSC #7636 [53], New Haven, CT, USA). It was routinely cultured in Lysogen Broth (LB) containing 10 g/L tryptone, 5 g/L yeast extract, and 10 g/L NaCl. A minimum of three biological repeats were included for each experimental condition.

Persister assays. Planktonic persister assays were carried out in 96-well, flask, or culture tube format as indicated. Seed culture was grown overnight (16 h) from a single colony on an agar plate. The overnight culture was used to inoculate subcultures (n = 3) with 100-fold dilution the next day for experiments, with incubation at 37 °C and shaking at 200 rpm. To determine persister counts, subcultures were washed and resuspended in PBS for treatment with ofloxacin at 10 µg/mL for 5 h, with shaking at 200 rpm, following the standard protocol [54]. After washing with PBS to remove antibiotics, a serial 10-fold dilution was performed and 10 µL drops were plated on LB agar plates to count the CFUs after incubation overnight at 37 °C.

Flow cytometry. Flow cytometry analyses were conducted using a BDAccuri C6 flow cytometer (BD Bioscience, Franklin Lakes, NJ, USA) at the Syracuse University Flow Core Facility. A total of 50,000 events were recorded for each sample (n = 3).

Fluorescent marker for monitoring bacterial growth. To monitor cell growth in real time, we tagged *E. coli* BW25113 with P*_rrnB_*_P1_-GFP_AGA_ chromosomally. Biparental mating was performed with the donor strain *E. coli* S17 λ *pir* carrying plasmid pDRXY22 (pUTminiTn5-Cm-*P_rrnB_*_P1_-*GFP_AGA_*) flanked by transposon ends following a previously described protocol [55]. The unstable *GFP_AGA_* tag enables the monitoring of cell growth through the activity of promoter *P_rrnBP1_*. Briefly, overnight cultures of both strains were mixed at a 1:1 ratio, resuspended in 20 μL LB medium, and dropped in the center of an LB agar plate for conjugational mating over 18 h at 37 °C. A streak of the bacterial mix was then resuspended in 5 mL 10 mM MgSO_4_, vortexed, diluted, and spread-plated onto select agar plates. The tagged strain was chemically transformed with the pRJW1 plasmid to obtain a *P_BAD_*-*hipA*-inducible strain with a GFP reporter to monitor growth activity at the promoter *P_rrnB_*_P1_.

Gene expression and analysis. Quantitative polymerase chain reaction (qPCR) was performed using SYBR green on a QuantStudio™ 3 Real-Time PCR System (ThermoFisher, #A28567). Thresholds were determined using pre-calibrated SYBR controls. The gene *rrsA* was used as housekeeping control. The forward and reverse primers (FP and RP, respectively) used for qPCR are listed in Table 1 for both *rrsA* and the gene of interest, *hipA*.

The target Ct values obtained were converted to fold difference expression ratios by normalizing with the empty host control and the *rrsA* housekeeping gene [56] using the PfaffI method for relative gene fold differences [57]. Standard curves for each gene investigated were generated in the empty host control for the calculation of efficiency values (E), per Equation (2):

(2) $$\mathrm{Fold}\;\mathrm{difference}=\frac{E^{∆C_{t\;target}}}{E^{∆C_{t\;normalizer}}},\;where\;E=10^{\frac{-1}{slope}}$$

RNA was isolated using the RNeasy kit (Qiagen, Germantown, MD, USA) following the manufacturer’s protocol for bacterial cultures. Two technical repeats were performed for each biological repeat (n = 3).

Microscopy and imaging analysis. An Axio Imager M1 fluorescence microscope (Carl Zeiss Inc., Berlin, Germany) was used to image cells. For cells without a fluorescent marker, Acridine Orange (AO) (ThermoFisher Scientific #A1301) staining was used to visualize the cells. Cells were washed and stained for 15 min in PBS in the dark. The total number of cells was counted using an etched hemacytometer (Hausser Dark-Line, Horsham, PA, USA).

Membrane potential. To further corroborate the results, we measured the membrane potential of cells using the DiOC2(3) dye (Invitrogen #B34950, Carlsbad, CA, USA) following manufacturer’s protocol, which aggregates when inside cells and emits red fluorescence when there is a hyperpolarized membrane [58]. The dye also emits green fluorescence in the monomeric form outside the cell, and therefore the ratio of red to green fluorescence provides a reliable measure of membrane potential [58].

Statistical analysis. One-way ANOVA analyses followed by the Tukey test were used for all statistical analyses using OriginPro, Version 2020b (OriginLab Corporation, Northampton, MA, USA). The *p* values are shown in the Results section as indicated. Results with *p* < 0.05 were considered statistically significant.

## Figures and Tables

**Table 1:** Primers for *hipA* and housekeeping gene *rrsA* for qPCR.

Gene	Primer Sequence
*rrsA*	FP: 5′-ctcttgccatcggatgtgccca-3′ RP: 5′-cagtgtggctggtcatcctctca-3′
*hipA*	FP: 5′-ggcggtacgggaatacacat-3′ RP: 5′-ccgggaatctcagcaccttt-3′

## Data Availability

The *E. coli* BW25113 utilized is the parent strain for the Keio Collection of single gene knockouts, obtained through the Coli Genetic Stock Center, as previously described [59].
